# Diversity of Mixotrophic Neutrophilic Thiosulfate- and Iron-Oxidizing Bacteria from Deep-Sea Hydrothermal Vents

**DOI:** 10.3390/microorganisms11010100

**Published:** 2022-12-30

**Authors:** Yang He, Xiang Zeng, Fei Xu, Zongze Shao

**Affiliations:** Key Laboratory of Marine Biogenetic Resources, Third Institute of Oceanography, Ministry of Natural Resources, Xiamen 361005, China

**Keywords:** deep-sea hydrothermal vents, mixotrophic, thiosulfate-oxidizing bacteria, iron-oxidizing bacteria, *Pseudomonas*, *Halomonas*

## Abstract

At deep-sea hydrothermal vents, sulfur oxidation and iron oxidation are of the highest importance to microbial metabolisms, which are thought to contribute mainly in chemolithoautotrophic groups. In this study, 17 mixotrophic neutrophilic thiosulfate- and iron-oxidizing bacteria were isolated from hydrothermal fields on the Carlsberg Ridge in the Indian Ocean, nine to the γ-proteobacteria (*Halomonas* (4), *Pseudomonas* (2), *Marinobacter* (2), and *Rheinheimera* (1)), seven to the α-proteobacteria (*Thalassospira*, *Qipengyuania*, *Salipiger*, *Seohaeicola*, *Martelella*, *Citromicrobium*, and *Aurantimonas*), and one to the Actinobacteria (*Agromyces*), as determined by their 16S rRNA and genome sequences. The physiological characterization of these isolates revealed wide versatility in electron donors (Fe(II) and Mn(II), or thiosulfate) and a variety of lifestyles as lithotrophic or heterotrophic, microaerobic, or anaerobic. As a representative strain, *Pseudomonas* sp. IOP_13 showed its autotrophic gowth from 10^5^ cells/ml to 10^7^ cells/ml;carbon dioxide fixation capacity with the δ13C_VPDB_ in the biomass increased from −27.42‰ to 3460.06‰; the thiosulfate-oxidizing ability with produced SO_4_^2−^ increased from 60 mg/L to 287 mg/L; and the iron-oxidizing ability with Fe(II) decreased from 10 mM to 5.2 mM. In addition, iron-oxide crust formed outside the cells. Gene coding for energy metabolism involved in possible iron, manganese, and sulfur oxidation, and denitrification was identified by their genome analysis. This study sheds light on the function of the mixotrophic microbial community in the iron/manganese/sulfur cycles and the carbon fixation of the hydrothermal fields.

## 1. Introduction

Iron and sulfur are elements widely present in the earth. The sources of iron and sulfur in the ocean include dust, coastal and shallow sediments, sea ice, and hydrothermal fluids [1]. Due to the perennial lack of light, the hydrothermal fluid ejected from the deep-sea hydrothermal vent contains a large amount of low-valent iron, manganese, reducing sulfide, methane, hydrogen, and other reducing compounds, which can be used as an electron donor for chemoautotrophic bacteria [2]. Additionally, heterotrophic γ-proteobacteria and α-proteobacteria were occasionally reported to have lithotrophic iron-, manganese-, and sulfur-oxidation capacities in some environments. Edwards et al. first reported that strains belonging to γ-proteobacteria (*Alcanivorax* sp., *Halomonas* sp., *Marinobacter* sp., and *Pseudomonas* sp.), α-proteobacteria (*Aurantimonas* sp., *Nitratireductor* sp. *Stappia* sp., and *Hyphomonas* sp.), and Actinobacteria (*Microbacterium* sp.) isolated from the eastern flank of Juan de Fuca Ridge off the coast of the Pacific Northwest were identified to have iron-oxidation and nitrate-reduction functions [3,4]. Representatives of six genera (*Metallogenium*, *Leptothrix*, *Siderocapsa*, *Naumaniella*, *Bacillus*, and *Pseudomonas*) in the phyla of Proteobacteria and firmicutes were also proven to participate in the oxidation of Fe(II) and Mn(II) in the bottom sediments of Lake Baikal in Russia [5]. Elemental iron and sulfur and amorphous iron sulfide enrichments derived from electrode biomass demonstrated products that were indicative of sulfur or iron oxidation by enrichment cultures belonging the genera *Halomonas*, *Idiomarina*, *Marinobacter*, and *Pseudomonas* of the γ-proteobacteria and *Thalassospira* and *Thioclava* from the α-proteobacteria [6]. Heterotrophic γ-proteobacteria (e.g., *Pseudoalteromonas* sp. and *Pseudomonas* sp.), isolated from a volcanic seamount located in the eastern-most island of American Samoa, were also reported to have the ability to catalyze ferrous iron oxidation under microaerobic conditions [7]. Despite the reports on the lithotrophic iron/manganese/sulfur-oxidizing ability of those heterotrophic Proteobacteria and firmicutes, little is known about these processes in hydrothermal vents. In this study, we isolated several neutrophilic thiosulfate- and iron-oxidizing bacteria, mainly thought as heterotrophic Proteobacteria, from deep-sea hydrothermal sulfides in Carlsberg Ridge in the Indian Ocean and further characterized their properties and possible molecular mechanisms.

## 2. Material and Methods

### 2.1. Sampling, Enrichment and Isolation

The samples were collected in the Wocan and Daxi hydrothermal vents, on the slowly expanding Carlsberg Ridge in the northwestern Indian Ocean on the DY38 cruise by the R/V Xiang Yang Hong 09, from 4 March to 23 March 2017. The crewed submersible Jiao Long collected samples of mussels, sulfides, hydrothermal sediments, and hydrothermal plumes around the active hydrothermal vents through five dives at several sites (6.4° N, 60.5° E; 6.8° N, 60.2° E; 3.7° N, 60.8° E), at a depth of 2928–3454 m (Table 1). Samples were stored in hermetically sealed sterile vials on board and transported at 4 °C to the laboratory. Oxygen- and Fe(II)-opposing gradient tubes were used with zero-valent iron and the released Fe(II) as the sole electron donor. The zero-valent iron plug at the bottom of the gradient tube provided the source of Fe(II). Sodium bicarbonate was used as the sole carbon source in the medium. Agarose-stabilized gradient tube cultures were set up with a zero-valent iron (ZVI) plug (1% *w*/*v* high-melt agarose) and semisolid medium (0.15% *w*/*v* low-melt agarose) [8]. Per 1000 mL of distilled water, the medium contained 27.50 g NaCl, 0.72 g KCl, 1.40 g CaCl_2_·2H_2_O, 5.38 g MgCl_2_, 6.78 g MgSO_4_·7H_2_O, 1.00 g NH_4_Cl, 10 mM NaHCO_3_, and 10 mM MES. After autoclaving, the medium was supplemented by a 1 mL/L trace mineral solution (https://www.atcc.org/products/md-tms, accessed on 25 December 2020) and a 1 mL/L vitamin solution (https://www.atcc.org/products/md-vs; www.atcc.org/products/md-tms, accessed on 25 December 2020) and was adjusted to pH 6.2 with CO_2_. The culture was enriched using agarose-stabilized gradient tubes at 28 °C [8,9]. When a yellow band appeared in the upper layer, a 200 μL medium was collected from the yellow band and then plated on a marine agar 2216 medium (BD Difco, San diego, CA, USA) at 28 °C for further isolation. After repeated plate streaking, the pure culture was obtained. These isolates were deposited in the Marine Culture Collection of China, MCCC (https://mccc.org.cn/, accessed on 25 December 2020) (Table 1).

### 2.2. Determination of Iron-Oxidation Capacity

The growths of isolated strains with a solid electron donor in the gradient tubes were tested. The varieties of electron donors include zero-valent iron, FeS, FeCO_3_, basalt, and iron-manganese nodules. FeCO_3_ and FeS were prepared as described by Hallbeck et al. [10] and Hanert et al. [11]. All reagents were chemically pure (CP) grade unless otherwise indicated. The basalt, pyrite, and iron-manganese nodules used are natural minerals from deep-sea environments, which were ground and sterilized for later use. The semisolid media with different electron donors were prepared separately. The strains were inoculated into the medium and cultured at 28 °C for up to 2 months.

Aerobic and anaerobic growths were also tested in aqueous media with oxygen or nitrate as electron acceptors. The preparation of stock solutions of FeCl_2_ was prepared as described by Emerson et al. [8]. The artificial seawater (ASW) in the iron oxidation-nitrate-reduction medium included 28.13 g NaCl, 0.77 g KCl, 1.60 g CaCl_2_·2H_2_O, 4.80 g MgCl_2_·6H_2_O, 0.11 g NaHCO_3_, 3.50 g MgSO_4_·7H_2_O, 0.001 g resazurin, and 1000 mL distilled water. The medium included 5 mM nitrate as the electron acceptor and was supplemented by 1/10,000 of yeast extract (Oxoid, Basingstoke, UK). Serum bottles with 50 mL volume were filled anoxically with 20 mL medium and were sealed with butyl rubber stoppers. The liquid in each serum bottle is gassed with a filter-sterilized N_2_:CO_2_ (80:20 *v*/*v*) gas mix for a minimum of 3 min. After autoclaving, the medium was supplemented by 10 mM NaHCO_3_, 1 mL/L Wolfe’s trace mineral solution, 1 mL/L vitamin solution, and 10 mM FeCl_2_·4H_2_O. The supplemented solution was sterilized by filtration through 0.22 μm Millipore filters. The anaerobic iron-oxidizing medium contained nitrate as the electron acceptor and FeCl_2_·4H_2_O as the energy source and reducing agents. The concentrations of nitrite produced divalent iron, and the total iron in the supernatant and that in the precipitate in the medium were measured.

### 2.3. Determination of Manganese-Oxidation Capacity

The growth in the K medium with 1 mM MnCl_2_ was added to test the ability of manganese oxidation. Per 1000 mL of distilled water, the medium contained 27.5 g NaCl, 0.05 g K_2_HPO_4_, 1.40 g CaCl_2_·2H_2_O, 1.00 g NH_4_Cl, 5.38 g MgCl_2_, 0.72 g KCl, 6.78 g MgSO_4_·7H_2_O, 2.0 g Peptone, 0.5 g yeast extract, and 4.766 g HEPES, and it was adjusted to pH 7.6 with NaOH. After autoclaving, the medium was supplemented by 1 mL sterilized MnCl_2_ (1 M) [12]. The strains were inoculated into K agar medium and cultivated for up to 20 days at 28 °C. The presence of Mn oxides around colonies was confirmed by using the leucoberbelin blue (LBB) assay [13].

### 2.4. Determination of Thiosulfate-Oxidizing Capacity

Growth in the autotrophic sulfur-oxidizing medium with sodium thiosulfate as the sole electron donor and sodium bicarbonate as the sole carbon source was observed, which was modified according to Lyu et al. [14]. The artificial seawater (ASW) in the SOB medium included 30.00 g NaCl, 0.14 g K_2_HPO_4_, 0.14 g CaCl_2_·2H_2_O, 0.25 g NH_4_Cl, 4.18 g MgCl_2_·6H_2_O, 0.33 g KCl, 0.005 g NiCl_2_·6H_2_O, and 1000 mL distilled water. After autoclaving, the medium was supplemented by sterilized 10 mL trace mineral solution, 10 mL/L vitamin solution, 12.5 mL NaHCO_3_ (8%), and 10 mL Na_2_S_2_O_3_ (1 M). Before inoculation, the cells were washed three times with sterilized seawater to exclude interference from organic carbon sources and then transferred to the autotrophic media three times. The strains were inoculated into the medium and cultured at 28 °C for 10 days, and the pH, thiosulfate, and sulfate concentrations were measured.

### 2.5. Determination of Carbon-Fixation Capacity

Carbon-fixation experiments were performed using autotrophic sulfur-oxidation medium with 10 mM NaH^13^CO_3_ as sole carbon source and sodium thiosulfate as sole energy source. Before inoculation, the organisms were washed three times with sterilized seawater to exclude interference from organic carbon sources and transferred three times. The strains were inoculated into the medium and cultured at 28 °C for 11 days. The pellets were lyophilized for 24 h and determined by Stable Isotope Ratio Mass Spectrometer (IRMS) (measuring accuracy = ±0.2‰; DeltaVAdvantage; Bremen, Germany). The measured method for δ13C‰ was referred to in Orcutt et al. [15]. The growth curve was determined by measuring the OD value of the culture medium, and the absorbance was tested at 600 nm by using a spectrophotometer (Varioskan LUX, Thermo Scientific, Waltham, MA, USA).

### 2.6. Measurements of Fe(II), Fe(III), Sulfate, and Nitrate

Ferrous iron was photometrically quantified with ferrozine after dilution in 1 M HCl [16]. Samples for total Fe analysis were reduced to the ferrous state with 200 mM hydroxylamine for 22–24 h [16].

Sulfate was detected by the barium-ion indication method [17] and the chromatography detection method by using an ion chromatograph (ICS1100, Thermo Scientific, Waltham, MA, USA) [18]. Nitrate and nitrite were detected via the sulfanilamide method [19].

### 2.7. Fluorescence Microscopy and Scanning Electron Microscopy/Energy Dispersive X-ray Spectroscopy (SEM/EDS)

The cells were observed using the LIVE/DEAD BacLight Bacterial Viability Kit (Invitrogen, Carlsbad, CA, USA), which contained SYTO 9 and propidium iodide [20], whose excitation/emission wavelengths were respectively set at 480/500 nm and 490/635 nm to perform dual-channel imaging for green and red fluorescence and further counted using a fluorescence microscope (Eclipse 80i, Nikon, Tokyo, Japan). The pellets were gently mounted on a 0.2 µm pore-size polycarbonate filter and air-dried to further examine them by using a FEI/Quorum PP3000T field-emission instrument (Quorum, Laughton, UK) and to analyze the elements by energy dispersive spectroscopy (EDS).

### 2.8. Phylogenetic Analysis of the Isolated Strains

The genomic DNA of isolated strains was extracted by the bacterial genomic extraction kit (Saibaisheng Co., Beijing, China). The complete 16S rRNA gene was amplified by PCR using bacterial universal primers: 27F (5′-AGAGTTTGATCCTGGCTCAG-3′) and 1492R (5′-ACGGCTACCTTGTTACGACT-3′) [21], and Sanger sequencing was performed. The multiple-sequence alignments were performed by ClustalW (Kyoto university, Kyoto, Japon) [22].The maximum-likelihood tree, based on the small-subunit (16S) rRNA gene, was constructed by IQtree v.1.6.12 (https://www.iqtree.org, accessed on 1 December 2022), with 1000 bootstraps using the “TIM3+F+I+G4” model chosen according to BIC [23]. Sequences of related taxa were obtained from the GenBank database (https://www.ncbi.nlm.nih.gov/genbank/, accessed on 1 August 2022 and EzBioCloud (https://www.ezbiocloud.net/, accessed on 1 August 2022) [24].

### 2.9. Genomic Analysis of the Isolated Strains

The genome sequences of isolated strains were determined by Shanghai Majorbio Bio-pharm Technology Co., Ltd. (Shanghai, China), using Solexa paired-end (500 bp library) sequencing technology. A total of 1 Gbp clean data with 300 × coverage was generated using the Hiseq 2000 platform (Illumina, San Diego, CA, USA). The clean data were assembled by SPAdes v.3.8.1 (http://cab.spbu.ru/software/spades/, (accessed on 20 January 2021) with the default settings [25]. The contigs longer than 1 kb and similar read coverage were kept for further analysis. SeqPrep (https://github.com/jstjohn/SeqPrep, accessed on 22 January 2021) and Trimmomatic [26] were used to check the Illumina library quality. Check M was used to check the genome quality [27]. Genome annotation was performed using the Rapid Annotation Subsystems Technology (RAST) server [28]. A whole genome BLASTX search was performed against the Kyoto Encyclopedia of Genes and Genomes (KEGG) (https://www.genome.jp/kegg/, accessed on 22 January 2021) [29], Clusters of Orthologous Groups (COG) (https://www.ncbi.nlm.nih.gov/research/cog/api/cog/, accessed on 22 January 2021) [30], Gene Ontology (GO) (http://geneontology.org/, accessed on 22 January 2021) [31] and Swiss-Prot databases (https://www.uniprot.org/, accessed on 22 January 2021) [32]. Further, rRNAs and tRNAs were predicted using barrnap (https://github.com/tseemann/barrnap, accessed on 10 February 2021) and tRNAscan-SE (http://trna.ucsc.edu/tRNAscan-SE/, accessed on 10 February 2021) [33], respectively. The average nucleotide identity (ANI) was calculated using the ANI calculator tool from EzBioCloud (https://www.ezbiocloud.net/tools/ani, accessed on 22 November 2022) [24]. The amino acid identity (AAI) values between the genomes of isolates and the reference strains of close genera were calculated using a web-based calculator available from (http://enve-omics.ce.gatech.edu/aai/, accessed on 22 November 2022) [34].

### 2.10. Nucleotide Sequence Accession Numbers for Strains

GenBank accession numbers of the 16S rRNA gene and genome for strains were deposited. They are shown in Table 1.

## 3. Results

### 3.1. Phylogenetic Affiliations

**Figure 1:**
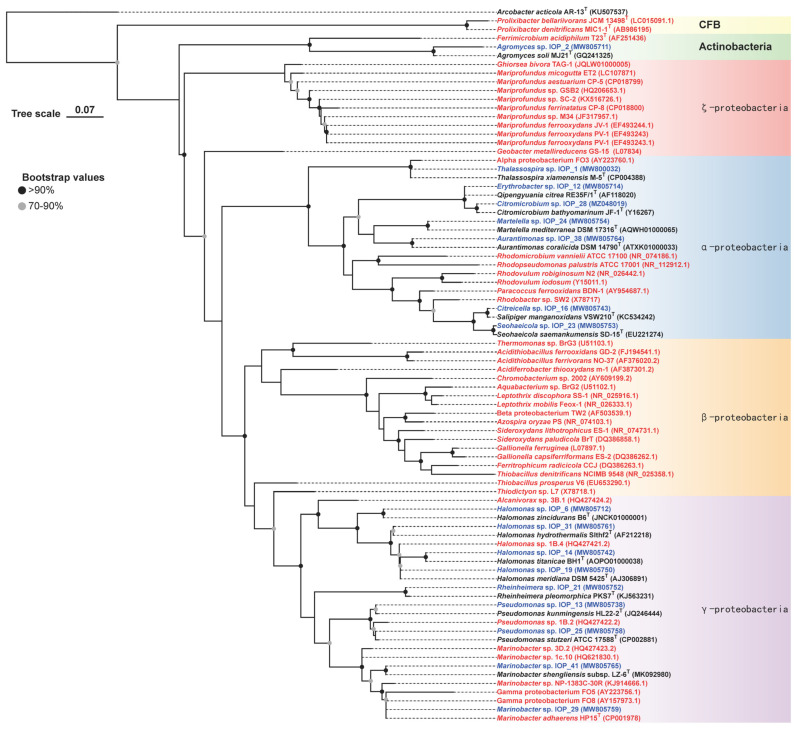
Phylogenetic relationships between cultured strains in this study (blue), reported autotrophic Fe oxidizers (red) and close relatives (black). The tree is a maximum-likelihood tree, based on the small-subunit (16S) rRNA gene (723 bp), showing bootstrap values (out of 1000 replicates).

Previous studies showed that most Fe-oxidizing bacteria (FeOB) belong to the bacterial phylum Proteobacteria, including the α-, β-, γ-, δ-, and ζ- classes [35]. Figure 1 shows the phylogenetic relationships among 17 strains in this study relative to other known representative FeOB. All the isolates in this study fell mainly within the α- and γ- classes of the Proteobacteria, except for strain IOP_2, belonging to the phylum Actinobacteria. Strains IOP_29 and IOP_41 were affiliated with *Marinobacter* (*M. adhaerens* HP15 and *M. shengliensis*). Some *Marinobacter* strains were reported to have neutrophilic iron-oxidizing capabilities from the in situ and lab enrichments of basalts, olivine minerals, etc. near hydrothermal vents, the subseafloor, and iron mine [4,6,36,37,38]; sulfur-oxidizing capabilities from marine sediments [37,39]; and Mn(II)-oxidizing capabilities from submarine basalts at Loihi seamount off the southeast coast of the island of Hawaii [40]. Four strains, namely IOP_6, IOP_14, IOP_19, and IOP_31, belong to the genus *Halomonas* by 16s rRNA gene analysis but with low ANI and AAI values (Supplementary Table S1). *Halomonas* spp. have been frequently identified as Mn(II)-oxidizing and Fe(II)-oxidizing bacteria from basalt in the Juan de Fuca Ridge flank, the volcanic Loihi seamount [36,40,41], the hydrothermal fields in Juan de Fuca Ridge flank, and [36,42] the acid mine drainage (AMD) environments in Southwest China [43]. Some *Halomonas* strains isolated from hydrothermal fluids and the sediment of hydrothermal field in the East Pacific Rise also showed sulfur-oxidizing capacity [44]. Strains IOP_13 and IOP_25 belong to genus *Pseudomonas*, which also has been frequently reported as iron-oxidizing bacteria. As described in Sudek et al. [7], *Pseudomonas* spp. had iron-oxidizing and Mn(II)-oxidizing capacities under microaerobic conditions from a volcanic seamount in the Juan de Fuca Ridge flank. *Pseudomonas* sp. FE13-26 extracted from sludge was also reported to efficiently oxide Fe(II) with extracellular enzyme ferroxidase [45]. *Pseudomonas* sp. LOB-2 was reported to have Mn(II)-oxidizing capacity isolated from submarine basalts at the Loihi seamount [40].

Additionally, seven isolates were affiliated to genera *Thalassospira*, *Qipengyuania*, *Salipiger*, *Seohaeicola*, *Martelella*, *Citromicrobium*, and *Aurantimonas* in the class α-proteobacteria (Figure 1, Table 2). Most of the iron-oxidizing α-proteobacteria were phototrophs, with the exception of *Paracoccus ferrooxidans* [46,47], which was a nitrate-dependent bacterium. Moreover, α-proteobacteria (*Aurantimonas* sp., *Nitratireductor* sp., *Stappia* sp., and *Hyphomonas* sp.) isolated from the eastern flank of Juan de Fuca Ridge were identified to have iron-oxidation and nitrate-reduction functions [4]. The genera *Thalassospira* and *Martelella* isolated from hydrothermal sulfides of the South Atlantic were previously also characterized to exhibit a sulfur-oxidizing ability [48]. Those results and our data all proved that some strains in α-proteobacteria had the ability of iron and/or sulfur oxidation to adapt to environmental conditions in deep-sea hydrothermal vents, which had not been notified before.

Strain IOP_2 was closest to *Agromyces soli* MJ21, with a 94.19% similarity in 16S rRNA genes. The iron-oxidizing ability was reported in organisms of the phylum Actinobacteria: *Microbacterium* sp. [3] and *Ferrimicrobium acidiphilum* [49]. The result of this study also showed the possible iron-oxidizing ability of other strains in phylum Actinobacteria.

### 3.2. Growth Test with Different Electron Donors and Acceptors

The growth tests with different electron donors and acceptors in gradient tubes and liquid medium under the aerobic, microaerobic, and anaerobic conditions for all strains are summarized in Table 2.

#### 3.2.1. Iron-Oxidizing Capacity

All the 17 isolates could oxidize FeS to form an iron-oxide layer in NCMA medium gradient tube (Supplementary Figure S1). Live/dead staining results showed that most of the cells of strain IOP_13, the same as other isolates, were alive in the iron-oxide layer (Supplementary Figure S2). Most isolates (13/17) could oxidize zero-valent iron (Table 2). Ten isolates, including all the *Halomonas* sp., *Pseudomonas* sp., *Marinobacter* sp., *Thalassospira* sp. IOP_1, and *Aurantimonas* sp. IOP_38, could oxidize FeCO_3_. Eight isolates, namely IOP_2, IOP_12, IOP_13, IOP_23, IOP_24, IOP_25, IOP_29, and IOP_38, could oxidize pyrite to iron oxides after 1 month. Notably, *Agromyces* sp. IOP_2 and *Halomonas* sp. IOP_6 could grow with basalt as the electron donor in gradient tubes.

**Figure 2:**
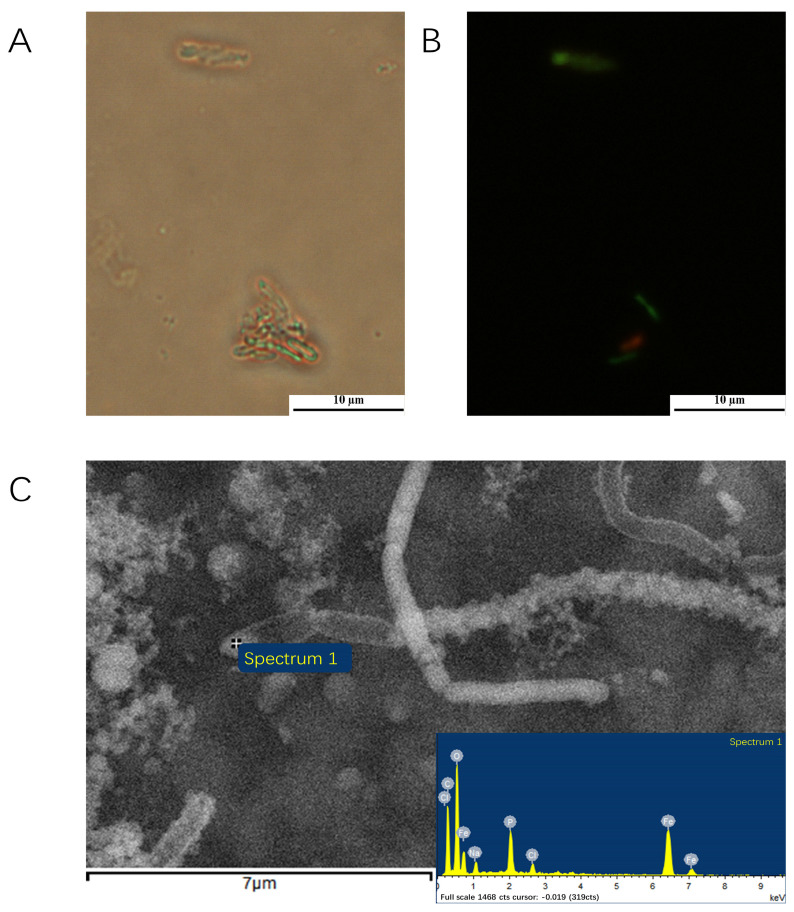
Light (**A**), fluorescence (**B**), and electron (**C**) microscopic images of strain *Pseudomonas* sp. IOP_13. Cells were grown anaerobically with ferrous chloride (FeCl_2_) and nitrate (NO_3_^−^).

**Figure 3:**
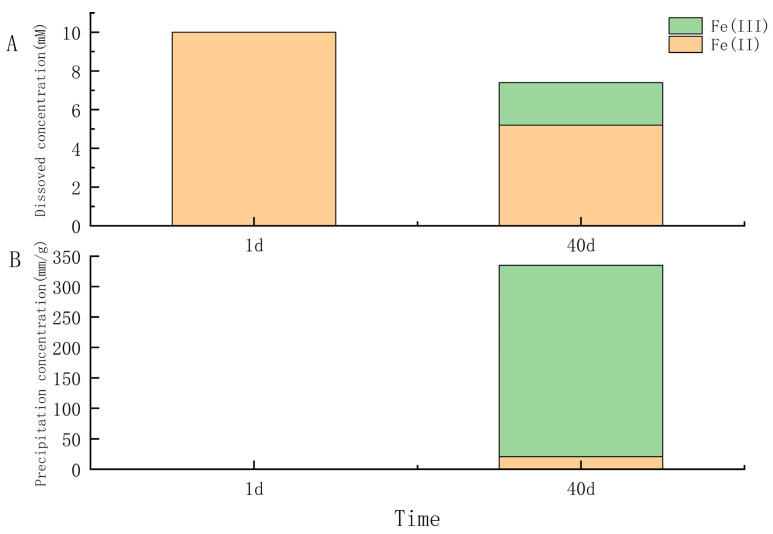
Fe(II) and nitrate consumption of *Pseudomonas* sp. IOP 13 in the presence of nitrate (10 mM) and ferrous iron over a period of 40 days. (**A**) Fe concentration in solution, (**B**) Fe concentration in precipitates.

Under anaerobic conditions, all nine γ-proteobacteria and four of seven strains in α-proteobacteria (*Salipiger*, *Seohaeicola*, *Martelella*, and *Citromicrobium*) could anaerobically grow with oxidizing FeCl_2_ and reducing nitrate. The growth of the cells, nitrite accumulation, and yellow-green precipitate after 40 days of all strains in anaerobic iron-oxidation media were examined. The results showed that under light and fluorescent microscopy, all the cells were observed to be encrusted with Fe(III) minerals, and some cells became dead with red fluorescent (Figure 2). Some isolates (IOP_6, IOP_14, IOP_19, IOP_31 in genus *Halomonas*; IOP_13 and IOP_25 in genus *Pseudomonas*; IOP_29, IOP_41 in genus *Marinobacter*; IOP_21 in genus *Rheinheimera*; IOP_16 in genus *Salipiger*; IOP_23 in genus *Seohaeicola*; IOP_24 in genus *Martelella*; and IOP_28 in genus *Citromicrobium*) produced yellow trivalent iron-oxide precipitates in the bottles during the process of cultivation. Nitrite is usually produced an intermediate product produced by bacteria using nitrate as an electron acceptor for anaerobic respiration. The accumulation of nitrite in those cultures was determined to be significantly higher (12.15 to 82.03 μM) than that in the negative control. This demonstrated that these strains use nitrate as an electron acceptor for anaerobic respiration. The nitrate reduction, nitrite formation, and Fe(II) oxidation were not observed in the noninoculated control medium. Strain *Pseudomonas* sp. IOP_13 produced significantly more nitrite than other strains and produced an orange trivalent iron-oxide precipitate after 10 days of growth (Supplementary Figure S3). The microscopy and EDS analysis of the strain *Pseudomonas* sp. IOP_13 showed that the cells were encrusted with minerals containing iron (Figure 2). The aqueous Fe(II) concentration in the culture decreased from 10 mM to 5.20 mM with dissolved Fe(III), which ranged from 0 mM to 2.2 mM after 40 days of growth (Figure 3A). The orange iron-oxide precipitate contained absorbed 20.63 mM/g Fe(II) and 314.58 mM/g Fe(III) (Figure 3B).

#### 3.2.2. Manganese-Oxidizing Capacity

Manganese-oxidizing bacteria can oxidize manganese chloride to manganese dioxide and produce dark-brown colonies on manganese-oxidizing plates. All isolates were cultured in a manganese-oxidation medium, and LBB assay was used on colonies cultured for 20 days. The redox stain (LBB) turned blue in the presence of the accumulation and conversion of manganese by strains *Pseudomonas* spp. IOP_13, IOP_25, and *Salipiger* sp. IOP_16.

Strain IOP_16 showed the capacity for Mn(II) oxidation; the closest organism to this strain was the manganese-oxidizing bacterium *Salipiger manganoxidans* VSW210, isolated from a shallow-water hydrothermal vent in Espalamaca (Azores) [50]. Two strains in the genus *Pseudomonas* (IOP_13 and IOP_25) that were capable of oxidizing Mn(II) were also identified. Supplementary Figure S4B showed the formation of a blue color on the colonies of strain IOP_13 after LBB staining. Orange halos around the colonies showed its siderphore-producing ability (Supplementary Figure S4A). Some *Pseudomonas* spp. were reported to have a potential of Mn(II)-oxidizing capacity. Kepkay and Nealson (1987) reported the occurrence of the growth of marine bacteria *Pseudomonas* sp. S-36, both mixotrophically on succinate or bicarbonate with Mn(II), in Mn-limited chemostats [51]. Mn(II)-oxidizing bacterium *Pseudomonas putida* GB-1 from a freshwater environment was also identified [52,53,54,55].

#### 3.2.3. Thiosulfate-Oxidizing Capacity

The growth and SO_4_^2−^ production of all strains in a thiosulfate oxidation medium, which contained an inorganic carbon source and sodium thiosulfate as an energy source, were examined. *Halomonas* spp. IOP_6, IOP_14, IOP_19, and IOP_31; *Pseudomonas* spp. IOP_13 and IOP_25; *Marinobacter* spp. IOP_29 and IOP_41 in γ-proteobacteria and *Thalassospira* sp. IOP_1; *Citromirobium* sp. IOP_28; and *Aurantimonas* sp. IOP_38 in α-proteobacteria showed an autotrophic sulfur-oxidizing capacity with monomeric sulfur and/or sulfate produced.

**Figure 4:**
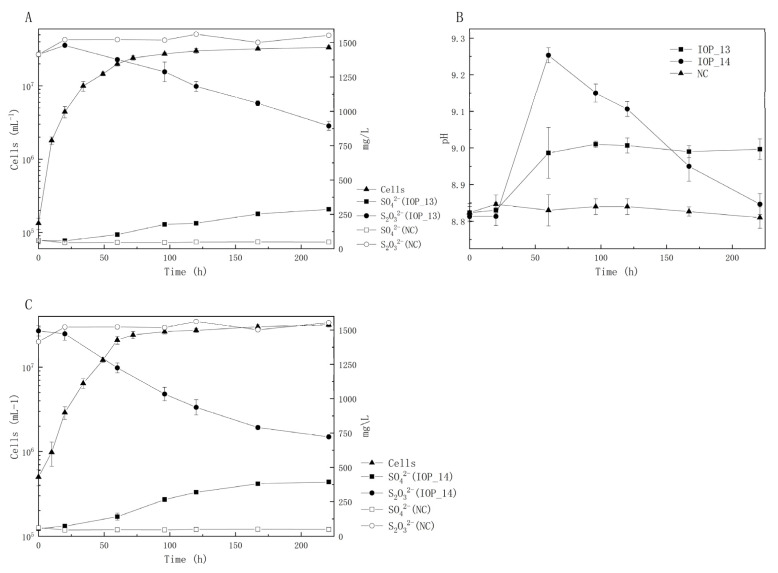
The growth of isolates in autotrophic sulfur-containing media. (**A**) the growth curve of *Pseudomonas* sp. IOP 13, (**B**) the growth curve of *Halomonas* sp. IOP_14, and (**C**) the pH change in cultures of strain IOP_13 and strain IOP_14.

The growth curves and the concentrations of electron donor S_2_O_3_^2−^ and product SO_4_^2−^ of strains *Pseudomonas* sp. IOP_13 and *Halomonas* sp. IOP_14 were further measured, as shown in Figure 4A,B. The results showed that the cell concentrations increased from the initial 10^5^ cells/mL to the final 10^7^ cells/mL after 8 days. The electron donor S_2_O_3_^2−^ was consistently consumed by strains IOP_13 and IOP_14 after inoculation and produced SO_4_^2−^ (287 mg/L, 394 mg/L) without SO_3_^2−^ detected. The pH value of the culture increased during the first 3 days (0.2 units and 0.5 units) and then stabilized (Figure 4C). This indicated that these strains produced alkaline substances in the process of sulfur oxidation.

### 3.3. Carbon Dioxide–Fixation Capacity

**Figure 5:**
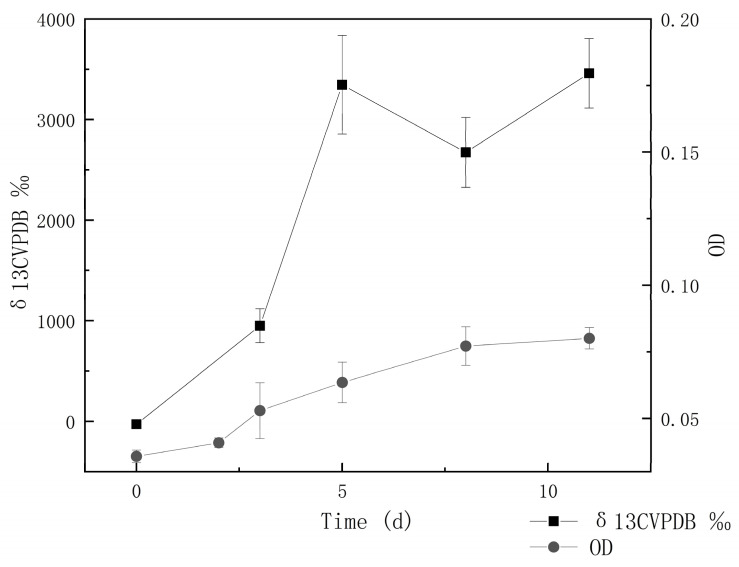
Growth curve and carbon-fixation capacity of *Pseudomonas* sp. IOP_13 grown in an autotrophic thiosulfate–containing medium.

All the strains were cultured in a sulfur-containing medium that used NaHCO_3_ as the sole carbon source. The results showed that 12/17 strains exhibited autotrophic growth. Strain IOP_13 was chosen to culture with NaH^13^CO_3_ as the sole carbon source to test its autotrophic capacity. The growth curve of strain IOP_13 and the value of δ13C in its biomass are shown in Figure 5. The results showed that bacterial cells increased from 10^5^ to 10^7^ cells/mL and that the δ13C_VPDB_ value of the bacterium increased from −27.42‰ to 3460.06‰ within 11 days, which indicated that strain IOP_13 utilized inorganic carbon as a carbon source.

### 3.4. Genome Characteristics of Isolated Strains

The 17 isolates were sequenced with 300 × coverage, and the draft genomes were produced with 98.69%–100.00% completeness (Supplementary Table S2). The genome similarities between isolates and closest relatives were also analyzed, with 76.50%–100.00% ANI values and 65.88%–99.95% AAI values (Supplementary Table S1).

**Figure 6:**
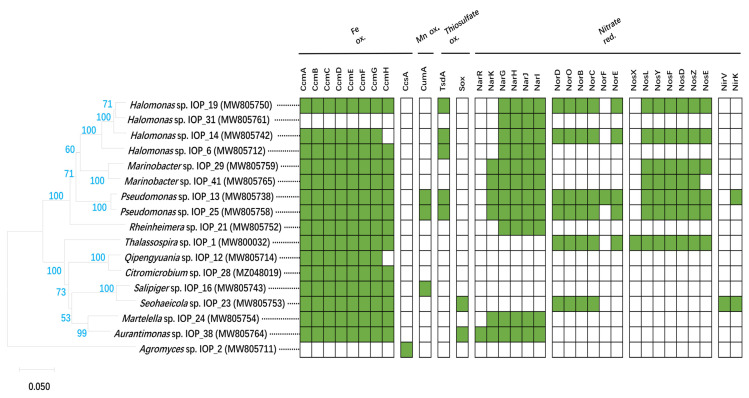
Functional gene annotations of the isolates in this study. Ccm, c-type cytochrome membrane proteins; CumA, multicopper oxidases; TsdA, tetrathionate-forming thiosulfate dehydrogenase; Sox, sulfur-oxidation gene (*SoxABCDHSFRSWXYZ*); Nar, nitrate reductase; Nor, nitric oxide reductase; Nir, nitrite reductase.

An analysis of the metabolic reconstruction using the KEGG database suggested that most of them have complete glycolysis, Entner–Doudoroff, pentosephosphate, and tricarboxylic acid cycle pathways, which showed their heterotrophic traits. The results for the functional gene annotation of strains are shown in Figure 6. Five strains, including *Halomonas* spp. IOP_6, IOP_14, and IOP_19 and *Pseudomonas* spp. IOP_13 and IOP_25, had high similarity with the sulfur-oxidation gene *TsdA* (Supplementary Figure S5). Tetrathionate-forming thiosulfate dehydrogenase (*TsdA*), identified by [56] from the purple sulfur bacterium *Allochromatium vinosum*, and this protein play the role of sulfur-based energy metabolism through the oxidation of thiosulfate. These isolates with the *TsdA* gene also showed a sulfur-oxidation ability, which could be carried out by the tetrasulphate pathway. *Aurantimonas* sp. IOP_38 and *Seohaeicola* sp. IOP_23 were annotated with the complete Sox sulfur-oxidation gene (*SoxABCDHSFRSWXYZ*). Strain IOP_38 could grow in an autotrophic sulfur-containing medium, but no sulfate or monosulfate production was detected. *Seohaeicola* sp. IOP_23 could grow only in a hetertrophic sulfur-containing medium.

The genes-encoding multicopper oxidases CumA [57] (Supplementary Figure S6) with the possible involvement in Mn(II) oxidation were found in strains IOP_13, IOP_16 and IOP_25, which have the capacity for Mn(II) oxidation.

Proteins thought to be involved in Fe(II) oxidation, namely *Cyc1* [58,59], *Cyc2* [58,59,60], *FoxABC* [61], *FoxEYZ* [62], *Sulfocyanin* [63], *MtoAB* [64], and *PioABC* [65], were queried against the genomes. A Fegenie analysis [66] showed that there were no known iron-oxidizing genes found in those isolates, in this study. The genomes were further searched for proteins containing the characteristic c-type cytochrome binding CXXCH motif [67]. Cytochromes c require the covalent attachment of heme via two thioether bonds at conserved CXXCH motifs, a process accomplished in prokaryotes by eight integral membrane proteins (CcmABCDEFGH). Heme is trafficked from inside the cell to outside (via CcmABCD) and chaperoned (holoCcmE) to the cytochrome c synthetase (CcmF/H) [68]. The results showed that 15 strains have possible c-type cytochromes maturation in systems I, except *Halomonas* sp. IOP_31 and *Agromyces* sp. IOP_2 (Figure 6). Strain IOP_2 contained *CcsA*, which belongs to the systems II pathway [67].

Some strains can couple iron oxidation with nitrate reduction. Denitrification-related genes were analyzed. Genes encoding the NarKGHJI, NirSKV, NorDQBCFE, and NosXLYFDZR proteins were annotated in 10 strains grown with iron oxidation and nitrate reduction. Enzymes NarGHI (nitrate reductase A) identified from *E. coli* [69] play the role of denitrification and support anaerobic growth on nitrate with a nonfermentable carbon source condition, and the genes encoding NarGHI were found in strains IOP_6, IOP_13, IOP_14, IOP_19, IOP_21, IOP_24, IOP_25, IOP_29, IOP_31, and IOP_41 (Supplementary Figure S7), but not IOP_38.

## 4. Discussion

### 4.1. Widespread of Mixotrophic Bacterial Strains in the Hydrothermal Vents

There is rich heterotrophic microbial life in hydrothermal vents. Versatile heterotrophic α- and γ-proteobacteria have been found in different venting areas of the Menez Gwen hydrothermal field in the Mid-Atlantic Ridge from the diffuse fluid discharge points through the mixing gradients to the plumes and the surrounding seawater [70]. Generalist species belonging to the genera *Marinobacter*, *Vibrio*, *Pseudoalteromonas*, *Halomonas*, *Pseudomonas*, and *Alcanivorax*, among others, have been repeatedly isolated from hydrothermal vent samples in the Pacific [4,71,72,73,74], and their abundance in vent fluids collected from the Pacific Ocean was estimated to be up to 28% of the total micro-organisms [75]. Phylotypes closely related to cultured species, e.g., *Alteromonas*, *Halomonas*, and *Marinobacter*, were relatively abundant in some crustal fluid samples in the Suiyo seamount off the eastern coast of Japan [76]. In this study, those heterotrophic α- and γ-proteobacteria were isolated from mussels, sulfides, hydrothermal sediments, and hydrothermal plumes around active hydrothermal vents (Table 1), which also indicated that they are widespread in these types of environments.

Compared with other Proteobacterial classes, ζ-proteobacterial populations as iron oxidizers have a narrow growth range and spread only in oxic-anoxic transition zones near shore environments [77] and iron-rich hydrothermal systems, such as iron-oxide material, hydrothermal sediment, etc. [78,79]. Until now, only seven species in two genera, *Mariprofundus* [80] and *Ghiorsea* [81], had been isolated, which have a narrow growth range of oxygen concentration in 0.07–2.0 μM [77]. Heterotrophic α- and γ-proteobacteria shown across broad environmental gradients and dominate in the hydrothermal plumes, sulfides, and sediments might have important roles in the element cycle of iron and sulfur, which have been under estimation before.

### 4.2. Diverse Metabolism of Mixotrophic Bacterial Strains in the Hydrothermal Vents

Theoretical studies demonstrate that mixotrophy is advantageous in oligotrophic or fluctuating environments [82]. Micro-organisms survive using an adaptation ability for effective competition in the hydrothermal ecosystem with shifting biogeochemical conditions [83,84]. For example, archaea *Pyrobaculum islandicum* from hydrothermal vents can live as heterotrophs or autotrophs strictly under piezophilic conditions. The SUP05 clade of gammaproteobacteria (Thioglobaceae) could act as abundant autotrophs in the hydrothermal fluids and in association with eukaryotes at hydrothermal vents or as heterotrophes throughout the ocean [85]. The heterotrophic genera *Pseudomonas*, *Halomonas*, and *Bacillus* were all reported to possess the tetrathionate-forming ability using thiosulfate as a supplemental inorganic energy source [86,87]. Many strains in the *Pseudomonas* genus, such as strains of *Pseudomonas stutzeri*, were shown to oxidize sodium sulfide, change thiosulfate into tetrathionate, or grow on a variety of substrates, such as FeCl_2,_ FeCO_3_ and FeSO_4_, as their sole energy source under anaerobically in the presence of nitrate [88,89,90]. We further predicted the sulfur-oxidizing and denitrification genes in 317 genomes belonging *Pseudomonas* in UniProt (http://sparql.uniprot.org/ accessed on 10 November 2022). It showed that 10 species, including *P*. *stutzeri* strain A1501, *P*. *aeruginosa*, *P*. *veronii*, *P*. *wadenswilerensis*, *P*. *reidholzensis*, *P*. *fluorescens*, *P*. *extremaustralis*, *P*. *marincola*, *Pseudomonas* sp. IsoF, and *Pseudomonas* sp. 9Ag-encoded TsdAB genes and NarI, were detected in 115 *Pseudomonas* genomes. It indicated that some *Pseudomonas* spp. have thiosulfate-oxidizing and nitrate-reducing potential for mixotrophic life. Some micro-organisms have been characterized as heterotrophes, but their autotrophic ability was not shown before. This study revealed their versatile metabolism as autotrophes and heterotrophes with sulfur and iron oxidization in aerobic-microaerobic-anaerobic conditions, and it further predicted possible mechanisms of these processes based on the annotation of their genomes. Sulfur oxidation with the tetrasulphate pathway by thiosulfate dehydrogenase was found common in *Halomonas* and *Pseudomonas* spp. of γ-proteobacteria, in this study. The thiosulfate-oxidizing enzyme (Sox) was found only in two isolates (*Seohaeicola* sp. IOP_23 and *Aurantimonas* sp. IOP_38) of α-proteobacteria. Almost all of them had genes for a complete pathway of denitrification. A total of 16 strains had c-type cytochromes maturation systems and c-type cytochromes with one or more heme-binding motifs, which may have a function in Fe redox reactions, as reported for iron oxidizers (*Rhodobacter* and *Sideroxydans* spp.) [62,64]. It is also noteworthy that *Agromyces* sp. IOP_2 in the phylum Actinobacteria with iron- and sulfur-oxidizing abilities lack all known iron- and sulfur-oxidizing genes, which needs to be further investigated.

However, it remains unknown how mixotrophic micro-organisms respond to different conditions across broad environmental gradients. The characterization of mixtrophs and their metabolisms deserve further attention.

## 5. Conclusions

With a rich variety of chemical energy sources and steep physical and chemical gradients, hydrothermal vent systems offer a range of habitats to support microbial life.

In this study, we isolated iron-oxidizing and thiosulfate-oxidizing bacteria from deep-sea hydrothermal vents and shed light on the potential diverse functions of these heterotrophic bacteria in γ- and α-proteobacteria and Actinobacteria. Based on their broad growth ranges and versatile metabolisms, we predicted that heterotrophic bacteria which have capacities of sulfur, iron, and manganese oxidation may play important roles in carbon, sulfur, and metal cycling in hydrothermal vents.

## Figures and Tables

**Table 1:** GenBank accession numbers of 16S rRNA genes and genomes for isolates in this study.

MCCC Deposition No.	Strains	Source	Closest Species	16S rDNA Identity (%)	16S rDNA GenBank Accession	Genome GenBank Accession
M28193	IOP_1	mussels	*Thalassospira xiamenensis* M-5(T)	98.79	MW800032	JAINWB000000000
MCCC 1A14002	IOP_2	mussels	*Agromyces soli* MJ21(T)	94.19	MW805711	JAINWC000000000
MCCC 1A14012	IOP_6	sulfides	*Halomonas zincidurans* B6 (T)	98.64	MW805712	JAIRBO000000000
MCCC 1A13998	IOP_12	hydrothermal sediments	*Qipengyuania citrea* RE35F/1(T)	98.78	MW805714	JAINWE000000000
M28194	IOP_13	hydrothermal sediments	*Pseudomonas kunmingensis* HL22-2(T)	98.61	MW805738	JAINWF000000000
M28195	IOP_14	plume	*Halomonas titanicae* BH1(T)	98.48	MW805742	JAINWD000000000
MCCC 1A13999	IOP_16	plume	*Salipiger manganoxidans* VSW210(T)	99.48	MW805743	JAINWH000000000
MCCC 1A14001	IOP_19	sulfides	*Halomonas meridiana* DSM 5425 (T)	98.82	MW805750	JAINWP000000000
MCCC 1A14003	IOP_21	sulfides	*Rheinheimera pleomorphica* PKS7 (T)	99.22	MW805752	JAINWG000000000
MCCC 1A14004	IOP_23	sulfides	*Seohaeicola saemankumensis* SD-15 (T)	99.04	MW805753	JAINWI000000000
MCCC 1A14005	IOP_24	sulfides	*Martelella mediterranea* DSM 17316 (T)	98.42	MW805754	JAINWJ000000000
M28196	IOP_25	sulfides	*Pseudomonas stutzeri* ATCC 17588(T)	98.55	MW805758	JAINWK000000000
MCCC 1A14006	IOP_28	sulfides	*Citromicrobium bathyomarinum* JF-1(T)	99.93	MZ048019	JAINWL000000000
MCCC 1A14007	IOP_29	sulfides	*Marinobacter adhaerens* HP15(T)	100.00	MW805759	JAINWM000000000
MCCC 1A14008	IOP_31	sulfides	*Halomonas hydrothermalis* Slthf2(T)	99.64	MW805761	JAIRBP000000000
MCCC 1A14010	IOP_38	sulfides	*Aurantimonas coralicida* DSM 14790(T)	99.93	MW805764	JAINWN000000000
MCCC 1A14013	IOP_41	sulfides	*Marinobacter shengliensis* LZ-6(T)	99.86	MW805765	JAINWO000000000

**Table 2:** Growth substrate tests of isolates in this study.

Strains	Most Similar Type Species	Iron Oxidiation						Manganese Oxidation	Sulfur Oxidation	
		Autotrophic-Microaerobic					Hetertrophic-Anaerobic	Heterotrophic	Autotrophic	Heterotrophic
		Fe^0^	FeS	FeCO_3_	Pyrite	Basalt	FeCl_2_	MnCl_2_	S_2_O_3_^2−^	S_2_O_3_^2−^
**Gammaproteobacteria**										
**IOP_6**	*Halomonas zincidurans* B6 (T)	+	+	+	−	+	+	−	+	−
**IOP_14**	*Halomonas titanicae* BH1(T)	+	+	+	−	−	+	−	+	+
**IOP_19**	*Halomonas meridiana* DSM 5425 (T)	+	+	+	−	−	+	−	+	+
**IOP_31**	*Halomonas hydrothermalis* Slthf2(T)	+	+	+	−	−	+	−	+	−
**IOP_13**	*Pseudomonas kunmingensis* HL22-2(T)	+	+	+	+	−	+	+	+	−
**IOP_25**	*Pseudomonas stutzeri* ATCC 17588(T)	+	+	+	+	−	+	+	+	−
**IOP_29**	*Marinobacter adhaerens* HP15(T)	+	+	+	+	−	+	−	+	+
**IOP_41**	*Marinobacter shengliensis* LZ-6(T)	+	+	+	−	−	+	−	+	−
**IOP_21**	*Rheinheimera pleomorphica* PKS7 (T)	−	+	−	−	−	+	−	−	+
**Alphaproteobacteria**										
**IOP_1**	*Thalassospira xiamenensis* M-5(T)	+	+	+	−	−	−	−	+	+
**IOP_12**	*Qipengyuania citrea* RE35F/1(T)	−	+	−	+	−	−	−	−	+
**IOP_16**	*Salipiger manganoxidans* VSW210(T)	+	+	−	−	−	+	+	−	+
**IOP_23**	*Seohaeicola saemankumensis* SD-15 (T)	+	+	−	+	−	+	−	−	+
**IOP_24**	*Martelella mediterranea* DSM 17316 (T)	+	+	−	+	−	+	−	−	+
**IOP_28**	*Citromicrobium bathyomarinum* JF-1(T)	−	+	−	−	−	+	−	+	−
**IOP_38**	*Aurantimonas coralicida* DSM 14790(T)	+	+	+	+	−	−	−	+	+
**Actinobacteria**										
**IOP_2**	*Agromyces soli* MJ21(T)	−	+	−	+	+	−	−	+	−
**NC1**		−	−	−	−	−	−	−	−	−
**NC2**		−	−	−	−	−	−	−	−	−

## Data Availability

Not applicable.

## Supplementary Materials

The following supporting information can be downloaded at https://www.mdpi.com/article/10.3390/microorganisms11010100/s1, Figure S1: Gradient tube (ZVI-based) growth of FeOB; Figure S2: Microscopic photo of bacterial cells from the iron-oxides layer in the gradient tube; Figure S3: Orange trivalent iron precipitate after 10 days growth for strain *Pseudomonas* sp. IOP_13 in nitrate-reduction and iron-oxidation medium; Figure S4: Colonies of strain *Pseudomonas* sp. IOP_13 growing on solid test media; Figure S5: Sequence alignment of TsdA proteins; Figure S6: Sequence alignment of CumA proteins in the isolates and CumA identified from *Pseudomonas putida* GB-1; Figure S7: Sequence alignment of Nar I proteins in the isolates and Nar I (respiratory nitrate reductase 1 gamma chain) identified from *E. coli*; Table S1: Average nucleotide identity (ANI) and average amino acid identity (AAI) between the genomes of isolates and their relatives; Table S2: Genomic characteristics of isolated strains.
