# Supplementary material for: Diversity of Mixotrophic Neutrophilic Thiosulfate- and Iron-Oxidizing Bacteria from Deep-Sea Hydrothermal Vents
Source: Microorganisms. 2022 Dec 30;11(1):100. doi: 10.3390/microorganisms11010100 (PMC9861301; doi:10.3390/microorganisms11010100)
Supplement: Supplementary file 1 [file microorganisms-11-00100-s001.zip › microorganisms-2058893-supplementary.pdf]

microorganisms

MDPI

Article

Diversity of Mixotrophic Neutrophilic Thiosulfate- and Iron-Oxidizing Bacteria from Deep-Sea Hydrothermal Vents

Yang He, Xiang Zeng *, Fei Xu and Zongze Shao *

Key Laboratory of Marine Biogenetic Resources, Third Institute of Oceanography, Ministry of Natural Resources, Xiamen 361005, China

* Correspondence: zengxiang@tio.org.cn (X.Z.); shaozz@163.com (Z.S.)

Supplementary Material

Supplementary Figures

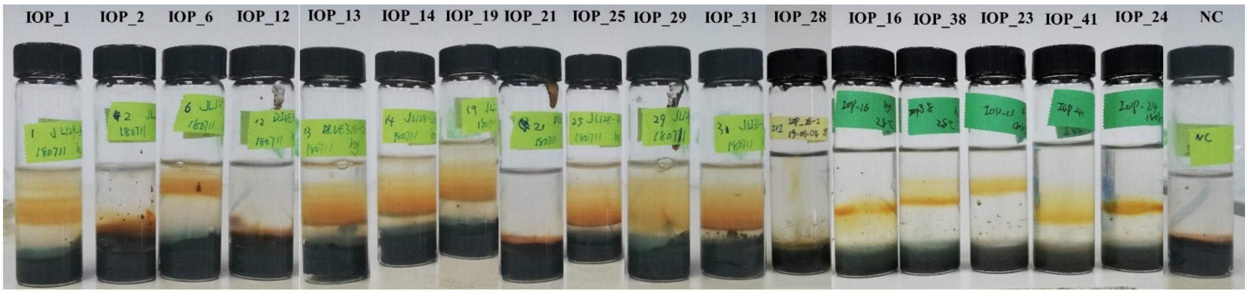

Figure S1. Gradient tube (ZVI-based) growth of isolates with iron-oxidizing ability.

A

B

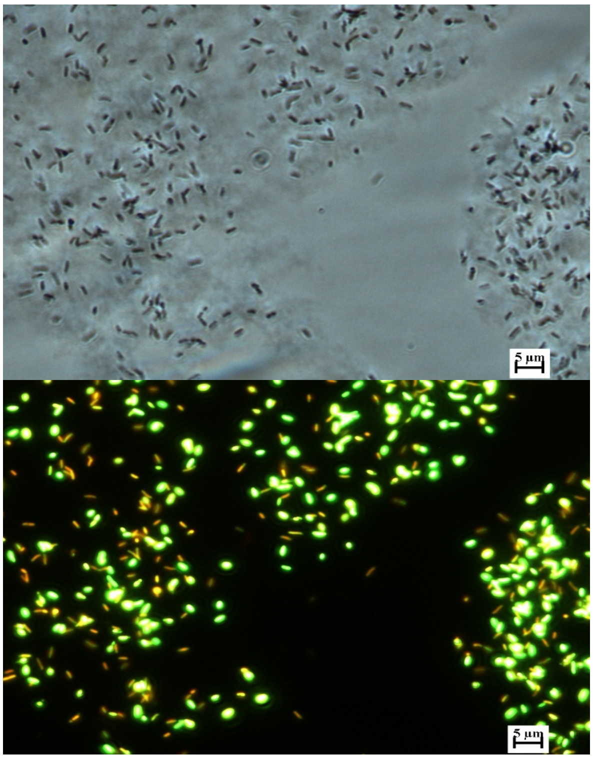

Figure S2. Microscopic photo of strain *Pseudomonas* sp. IOP_13 from the Iron-Oxides layer in the gradient tube. (A) bacterial cells under light microscopy; (B) Live cells are green stained, and dead cells are red stained by LIVE/DEAD™ BacLight™ Bacterial Viability Kit, which contained SYTO 9 and propidium iodide.

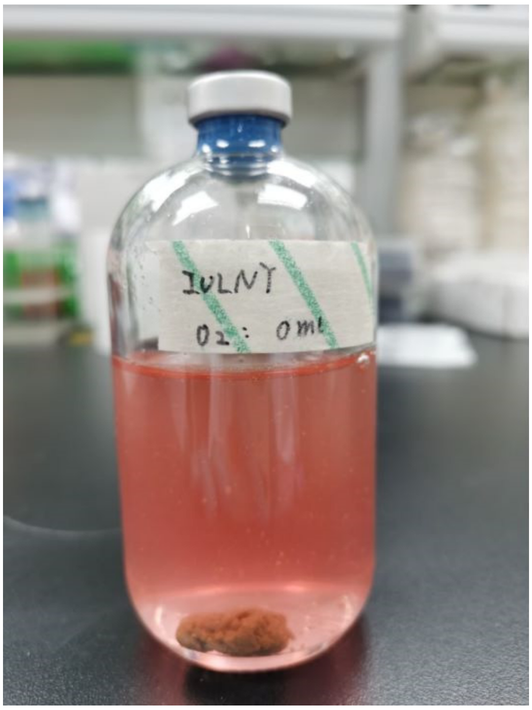

Figure S3. Orange-coloured trivalent iron precipitate after 10 days growth for strain *Pseudomonas* sp. IOP_13 in nitrate reduction-iron oxidation medium.

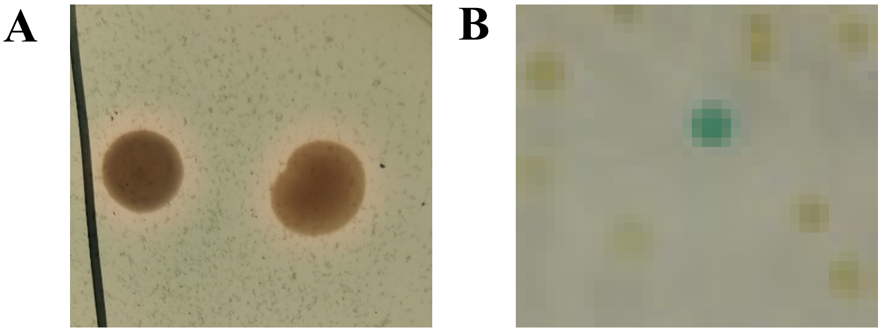

Figure S4. Colonies of strain *Pseudomonas* sp. IOP_13 growing on solid test media. (A) Orange halos around the colonies of strain IOP_13 on CAS agar plates. (B) Detection of manganese accumulation by strain *Pseudomonas* sp. IOP_13 using leucoberblin blue (LBB) spot test.

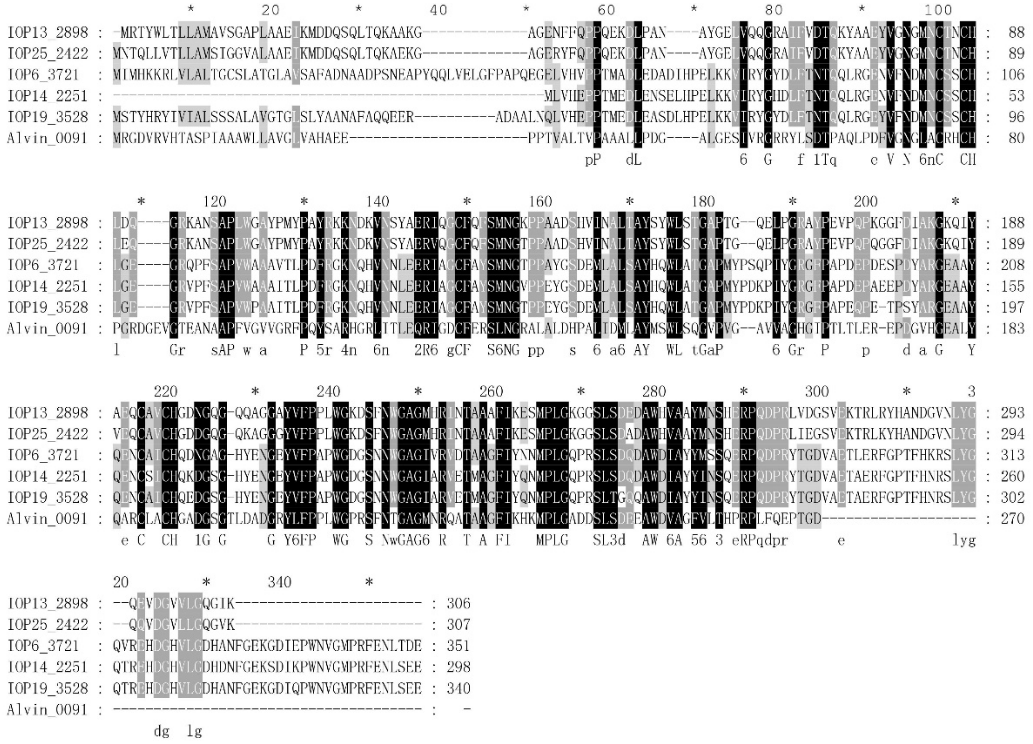

Figure S5. Sequence alignment of TsdA proteins. Species: IOP_13 (IOP13_2898), IOP_14 (IOP14_2251), IOP_6 (IOP6_3721), IOP_25 (IOP25_2422), IOP_19 (IOP19_3528), *Allochromatium vinosum* DSM 180T (Alvin_0091, accession: ADC61061).

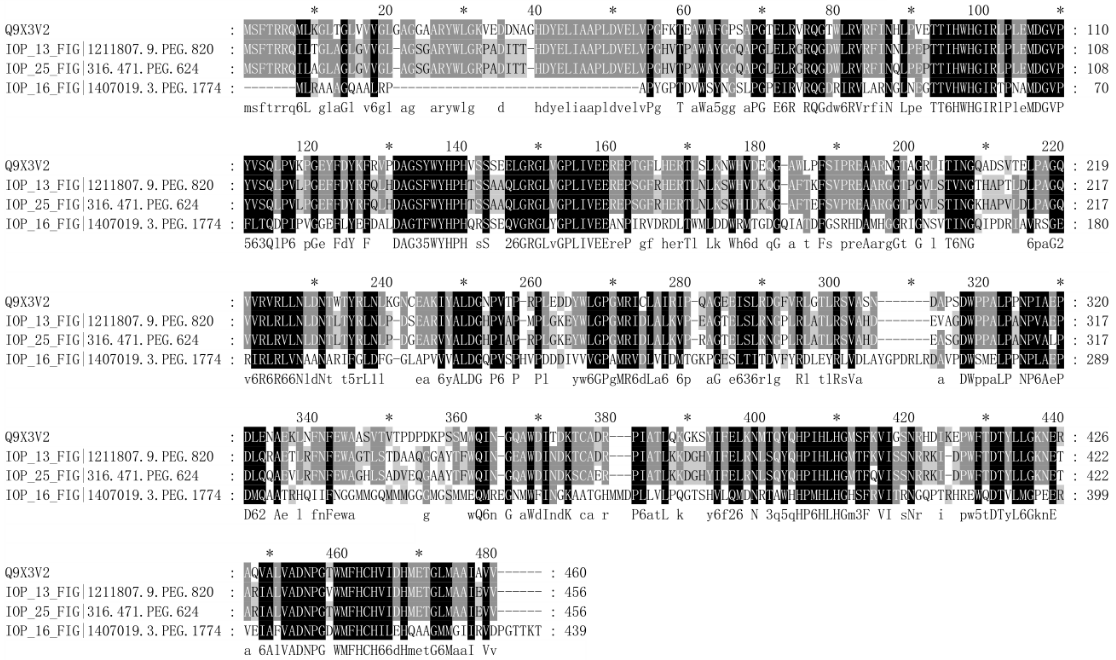

Figure S6. Sequence alignment of CumA proteins in the isolates and CumA identified from *Pseudomonas putida* GB-1. Species: *Pseudomonas putida* GB-1 (accession: Q9X3V2), IOP_13 (IOP13_820), IOP_25 (IOP25_624), IOP_16 (IOP16_1774).. Strictly conserved residues are marked with black background.

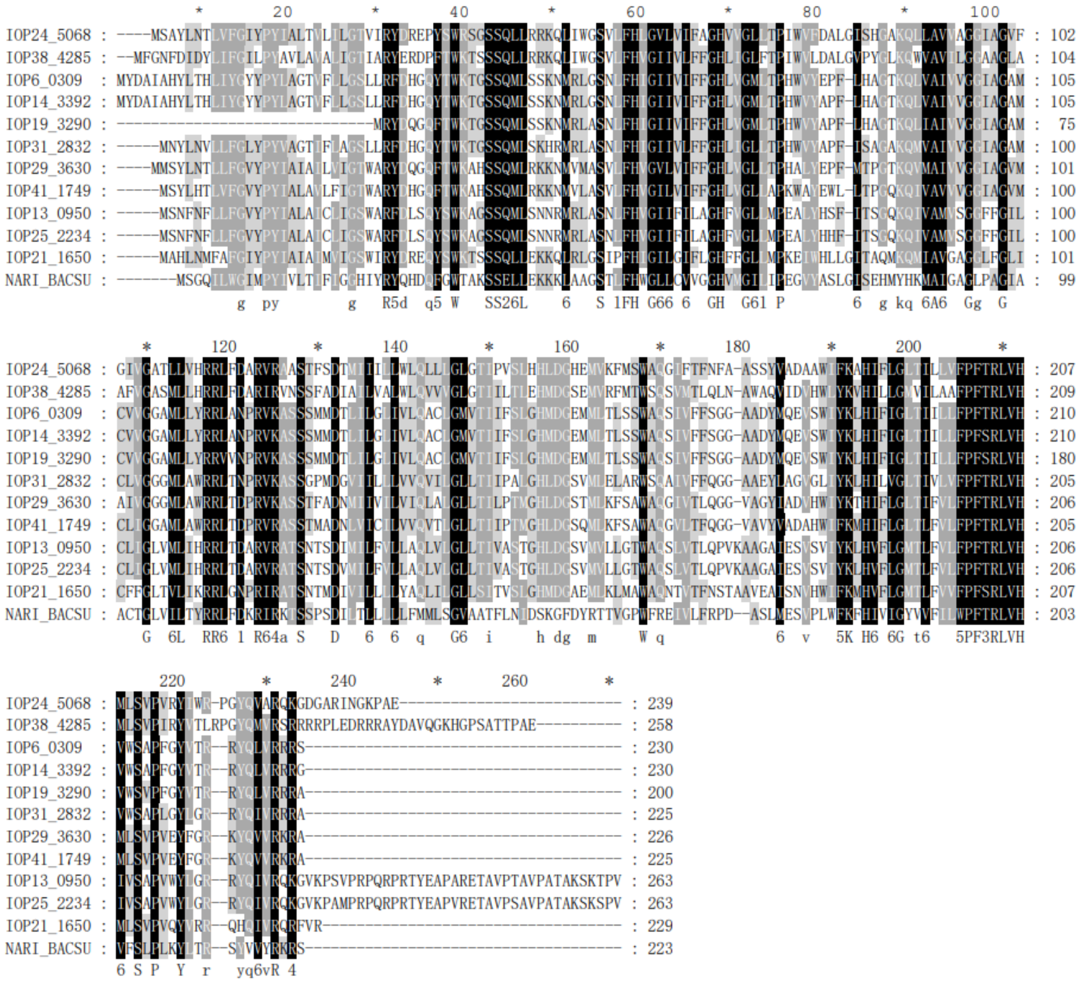

Figure S7. Sequence alignment of Nar I proteins in the isolates and Nar I (Respiratory nitrate reductase 1 gamma chain) identified from *Bacillus subtilis*. Species: IOP_24 (IOP24_5068), IOP_38 (IOP28_4285), IOP_6 (IOP6_0309), IOP_14 (IOP14_3392), IOP_19 (IOP19_3290), IOP_31 (IOP31_2832), IOP_29 (IOP29_3630), IOP_41 (IOP41_1749), IOP_13 (IOP13_0950), IOP_25 (IOP25_2234), IOP_21 (IOP21_1650), *Bacillus subtilis* K12 (NARI_ECOLI, accession: P11350). Strictly conserved residues are marked with black background.

Supplementary Tables

Table S1. Average Nucleotide Identity (ANI) and Average Amino acid Identity (AAI) between the genomes of isolates and their relatives.

Strain	Reference genomes compared	ANI	AAI
IOP_1	*Thalassospira xiamenensis* M-5	100.00%	99.94%
IOP_2	*Agromyces soli* KACC 16215	92.79%	94.66%
IOP_6	*Halomonas zincidurans* B6	76.90%	65.88%
IOP_12	*Qipengyuania citrea* CGMCC 1.8703	98.09%	98.19%
IOP_13	*Pseudomonas kunmingensis* HL22-2	97.00%	97.29%
IOP_14	*Halomonas titanicae* BH1	97.57%	97.58%
IOP_16	Salipiger marinus DSM 26424	98.24%	98.06%
IOP_19	*Halomonas meridiana* DSM 5425	97.28%	96.60%
IOP_21	*Rheinheimera pleomorphica* PKS7	79.91%	81.32%
IOP_23	*Seohaeicola saemankumensis* DP4N-5	76.50%	65.89%
IOP_24	*Martelella mediterranea* DSM 17316	93.63%	93.88%
IOP_25	*Pseudomonas stutzeri* ATCC 14405	86.07%	90.15%

IOP_28	*Citromicrobium bathyomarinum* JL354	100.00%	99.95%
IOP_29	*Marinobacter adhaerens* HP15	87.62%	93.52%
IOP_31	*Halomonas hydrothermalis* Slthf2	77.08%	65.90%
IOP_38	*Aurantimonas coralicida* DSM 14790	96.30%	97.31%
IOP_41	*Marinobacter shengliensis* LZ-6	96.04%	96.31%

Table S2. Genomic characteristics of isolates.

Strain	Genome size (bp)	Contigs	G+C content (mol%)	L50	N50	Completeness	Contamination
IOP_1	4808071	24	54.8	4	434721	100	0.00
IOP_2	3872332	5	72.2	1	1975501	99.33	0.67
IOP_6	3989131	26	52.6	4	478224	99.43	0.95
IOP_12	3247055	19	63.9	3	535616	99.48	0.42
IOP_13	4998573	159	62.1	6	282269	100	0.14
IOP_14	5280855	63	54.7	9	171700	99.57	0.89
IOP_16	5034081	72	66.6	6	292303	99.10	1.53
IOP_19	3688912	234	56.8	10	90895	100	0.44
IOP_21	4301081	14	49.5	2	673391	100	0.22
IOP_23	4115415	44	60	5	329396	98.69	0.28
IOP_24	5424777	69	62.2	12	131375	99.03	0.32
IOP_25	4545959	41	63.6	4	430176	100	0.14
IOP_28	3213860	3	65	1	2874297	99.93	0.00
IOP_29	4478111	12	57.1	2	796908	100	0.00
IOP_31	3688243	89	64.2	13	95977	99.07	0.11
IOP_38	4734847	62	66.6	4	448666	100	0.00
IOP_41	4305604	84	57.1	9	151162	100	0.05
